# Temperatures Influence Susceptibility to Insecticides in *Aedes aegypti* and *Aedes albopictus* (Diptera: Culicidae) Mosquitoes

**DOI:** 10.3390/pathogens10080992

**Published:** 2021-08-06

**Authors:** Wendy S. Salinas, Teresa P. Feria-Arroyo, Christopher J. Vitek

**Affiliations:** Center for Vector-Borne Disease, Biology Department, The University of Texas Rio Grande Valley, 1201 West University Dr, Edinburg, TX 78539, USA; wendysalinas22@gmail.com (W.S.S.); teresa.feriaarroyo@utrgv.edu (T.P.F.-A.)

**Keywords:** *Aedes aegypti*, *Aedes albopictus*, insecticide resistance, mosquito control

## Abstract

*Aedes aegypti* and *Aedes albopictus* (Diptera: Culicidae) are vectors for several arboviruses, including dengue, Zika virus and chikungunya virus. The primary method of controlling these diseases is controlling the vector population, often with insecticides. Insecticide resistance may impact the success of these efforts. We tested the effect of variable temperature exposures on susceptibility to insecticides by exposing adult *A.* *aegypti* and *A. albopictus* to different temperatures and tested their susceptibility to insecticides. We hypothesized that adults maintained at high temperatures would show increased susceptibility to insecticides relative to lower temperatures. Colony mosquitoes were hatched, reared to adulthood and then maintained in three temperature regimes that reflect average seasonal temperatures in the Rio Grande Valley, TX. Susceptibility to permethrin and deltamethrin was assessed using the CDC bottle bioassay method. Overall *Aedes albopictus* had higher susceptibility to all insecticides than *Aedes aegypti*. Mosquitoes kept at different temperatures exhibited differential susceptibility to insecticides. Low temperature exposed mosquitoes had decreased susceptibility while high temperature conditions resulted in increased mortality. Our results suggest public health officials must consider temperature effects when controlling mosquitoes with insecticides.

## 1. Introduction

The re-emergence of vector-borne diseases highlights the increased need for effective vector-borne disease control measures. Both *Aedes aegypti* and *Aedes albopictus,* vectors of multiple vector-borne diseases, are found in the Rio Grande Valley (RGV) in South Texas, along the transboundary region Mexico–US [1,2]. One effective control effort for these diseases is the use of insecticides to control the vector populations. This control method has historically been effective in keeping mosquito populations low [3]. However, the evolution of insecticide resistance may be one of several factors resulting in the increased spread of vector-borne diseases [4,5].

Concerns about insecticide resistance have commonly focused on genetic mutations that allow the mosquitoes to survive exposure to the insecticide [6]. Target site mechanisms, such as the *kdr* mutation that occurs in the voltage gated sodium channel gene, prevent uptake of insecticides. Detoxification mechanisms, such as esterase detoxification, are another method of resistance and consist of processes that allow mosquitoes to break down insecticides. Studies examining field populations of mosquitoes often assess the presence of these or related mechanisms to assess resistance [7]. However, it is likely that environmental variables, such as temperature, may also influence the efficacy of insecticides. Previous research has shown strong links between temperature, physiology, and life history [8,9,10,11,12,13]. Some studies suggest that temperature may influence susceptibility to insecticides in *A. aegypti* adult mosquitoes reared in the field [14,15]. These influences on susceptibility may not be detected simply through genetic analysis but must be tested in different relevant conditions.

The RGV is a sub-tropical climate with suitable weather for mosquitoes year-round [16,17]. South Texas experiences hot summers, a relatively warm spring and fall, and mild winters. We hypothesized that temperature would influence a mosquito’s susceptibility to insecticides in RGV populations. To test this, we used recently generated field colonies of local mosquito species, *A. aegypti* and *A. albopictus*, and maintained the adult populations at one of three temperature regimes prior to testing exposure to insecticides. The temperatures used reflect typical seasonal temperatures in the RGV. We predicted that higher susceptibility would be observed at summer temperatures in both species due to the potential physiological stress imposed by temperatures greater than 35 °C.

## 2. Results

**Figure 1:**
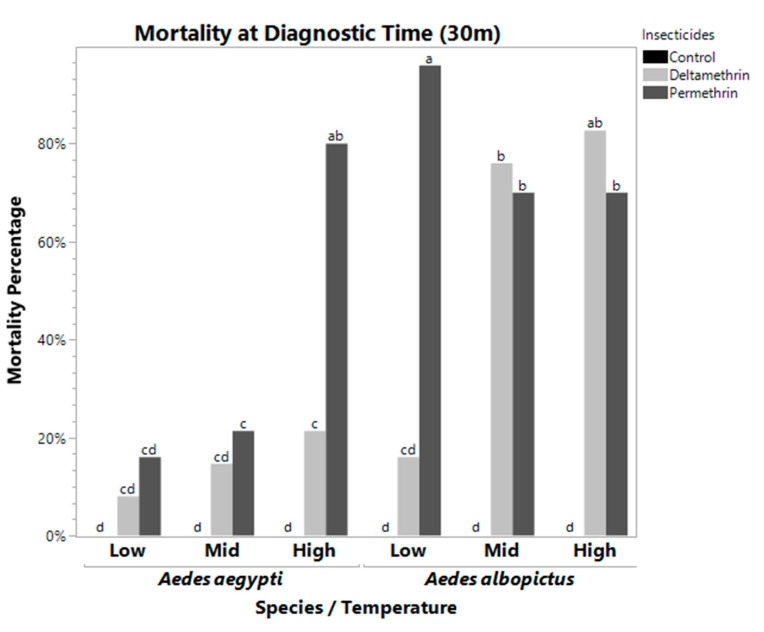
Mortality Rate at Diagnostic Time for *Aedes aegypti (left)* and *Aedes albopictus (right).* These bars represent the mean mortality percentage for all bottles of 25 mosquitoes. Bars with the same letters are not significantly different from each other. At low temperatures, deltamethrin and permethrin were significantly different from each other in *Aedes albopictus*, which was not observed at higher temperatures. Mortality in *Aedes aegypti* was reduced at all temperatures and with all insecticides, in some cases no different from a control, with the exception of high temperature permethrin treatment.

The ANOVA examining mortality differences influenced by insecticide, species, and temperature was significant at both the diagnostic time (DT) (F = 45.7056, df = 4, *p* ≤ 0.0001) and the final 120 min time (F = 10.8605, df = 4, *p* ≤ 0.0001). The individual effects were significant at the DT (Table 1 and Table 2). At the DT *A. albopictus* had a significantly higher mortality (43.68% ± 8.07%) when exposed to insecticides than *A. aegypti* (17.93% ± 4.68%) (F = 232.6249, df = 1, *p* ≤ 0.0001). Overall, permethrin was a more effective insecticide (F = 253.2861, df = 2, *p* ≤ 0.0001), with increased mortality (Figure 1). Lastly, high temperatures resulted in significantly increased mortality relative to mid and low temperatures (F = 40.0163, df = 2, *p* ≤ 0.0001). Similar results were observed at 120 m (Table 2) with similar significance levels (Table 1).

The two-way interactions were significant at both the DT and 120 m, except for species*temperature at 120 m (Table 1). There is a clear differential response between the species for mid temperature permethrin and deltamethrin, low temperature permethrin, and high temperature deltamethrin. Using a Tukey–Kramer HSD post-hoc test, we identified which treatment pairs were significantly different from each other. Bars with the same letter are not significantly different from each other. At the DT, temperature interacted with species (F = 9.6182, df = 2, *p* = 0.0005). *Aedes aegypti* at high temperature had significantly higher mortality than mid and low temperatures. However, *A. albopictus* was not significantly different to *Aedes aegypti* at low and mid temperature (Figure 1). Temperature also significantly interacted with insecticide (F = 26.1255, df = 4, *p* ≤ 0.0001), suggesting mosquito susceptibility varied based on temperatures at which adults were maintained following pupal eclosion. Permethrin was not significantly different at mid and high temperatures, but the low temperature treatment had decreased mortality. In addition, species also significantly interacted with insecticides (F = 60.6902, df = 2, *p* ≤ 0.0001).

**Figure 2:**
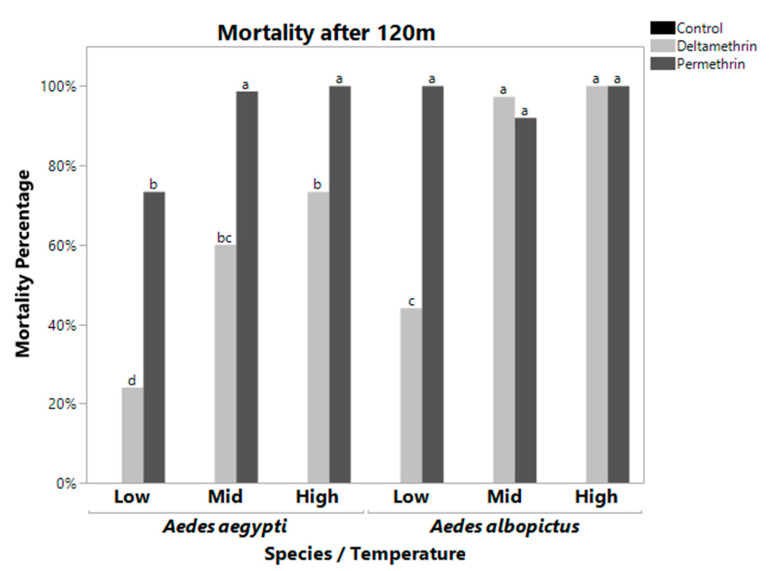
Mortality Rate at 120 m for *Aedes aegypti (left)* and *Aedes albopictus (right)*. These bars represent the mean mortality percentage for all bottles of 25 mosquitoes. Bars with the same letters are not significantly different from each other. Controls were significantly different from all treatments. *Aedes albopictus* had close to 100% mortality for all treatments except low temperature deltamethrin. *Aedes aegypti* only had 100% mortality for mid and high temperature permethrin. *Aedes aegypti* had significantly reduced mortality at all temperatures when exposed to deltamethrin, while this was observed in *Aedes albopictus* only at the low temperature treatment.

Similar patterns were observed at 120 m although with overall higher levels of mortality (Figure 2). At 120 m, temperature significantly interacted with species (F = 2.3748, df = 2, *p* = 0.1083). Within *A. aegypti*, high and mid temperature responses were different to low temperature response. *Aedes albopictus* at low temperature was significantly different than *A. albopictus* at high and mid temperatures (Figure 2). Temperature also significantly interacted with insecticide (F = 57.6673, df = 4, *p* ≤ 0.0001). Low temperatures showed decreased mortality rates with the exception of permethrin in *Aedes albopictus*. In addition, species also significantly interacted with insecticides (F = 41.6820, df = 2, *p* ≤ 0.0001).

There was also a significant three-way interaction between species, temperature, and insecticide (Table 1). Table 3 shows each treatment combination with the mean mortality ± the standard error. Treatments with the same significance letters indicate that they are not significantly different from each other (excluding controls which had 100% survivorship). Treatment with 100% average mortality has a standard error of zero. At the DT and 120 m, the three-way interaction was significant (F = 45.7056, df = 4, *p* < 0.0001 for DT, F = 10.8605, df = 4, *p* < 0.0001 for 120 m, Table 1). At the DT, *A. aegypti* displayed low mortality rates when exposed to deltamethrin and permethrin at low temperatures, but the mortality rate jumped significantly when exposed to permethrin when maintained at high temperatures. *Aedes albopictus* had constantly high mortality to permethrin at all three temperature treatments (although it dropped significantly at mid and high temperature treatments), while mortality due to deltamethrin was significantly lower at low temperature exposures than mid and high temperature exposures (Figure 1, Table 3). At 120 m, *Aedes albopictus* had 100% mortality to permethrin at low, mid, and high temperature treatments, and 100% mortality to deltamethrin at mid and high temperature treatments. *Aedes aegypti* mortality was 100% for permethrin only at mid and high temperature.

## 3. Discussion

Insecticide resistance studies are routinely conducted to monitor changes in susceptibility in mosquito populations [13,14,15,18,19] but only a few studies have tested how temperature may influence insecticide susceptibility on arthropods [3,13,19,20,21,22]. One such study indicated that temperature influences susceptibility to insecticides in *A. aegypti* mosquitoes in several areas of the world although they did not vary temperature for day/night differences, and only tested as low as 28 °C. [22]. Research conducted in Egypt with *Culex pipiens* mosquito species showed similar results to this study, suggesting that mosquitoes at lower temperatures had a decreased susceptibility to insecticides compared to mosquitoes which were exposed to high temperatures [13]. However, that study’s highest temperature of mosquito exposure was 30 °C, which is lower than summer temperatures in the RGV.

Changes in ambient temperatures may also affect metabolism, binding-affinity, andchemical up-take in insects [23,24]. Exposure to daily variation in temperature (mimicking “natural” day/night variation) influence upregulation of heat shock proteins and heat tolerance in mosquitoes [25]. This may also influence metabolic resistance and the ability to detoxify insecticides. Normally, exposure to pyrethroids and organochlorines causes overstimulation of the mosquito’s nervous system [26]. Gene expression of the *kdr* mutation may reduce sensitivity of the sodium gated channels [27] and may also be a mechanism by which susceptibility may vary and should be explored.

In our study, adult mosquitoes were exposed to varying temperatures for a relatively short period of time (5 to 10 days) following pupal emergence. Despite this short exposure time, our data indicates a significant difference in susceptibility to insecticides. Longer exposure to temperatures may increase or alter the differential response to insecticides, although long term maintenance of colonies at the “high” temperature may be difficult due to higher mortality rates. Rearing larvae at different temperatures may further alter the results of this study and should be examined as a potential factor. The use of F4 generation eggs for low temperature *Aedes aegypti* (as indicated in the methods) did not seem to produce any discrepancy in the data.

In the lower RGV area of Texas, the primary insecticide used for control efforts is permethrin (in many cases, the only insecticide). Control efforts are primarily conducted during spring, summer, and fall months as needed based on nuisance calls or disease outbreaks. Adult control consists of spraying commercially purchased permethrin-based insecticide from ground-based vehicles. Previous studies testing insecticide susceptibility in field populations of mosquitoes have suggested that there is seasonal variability in susceptibility even if mosquitoes are exposed to identical conditions [28]. Our data, combined with the observed seasonal variability, suggest that susceptibility patterns are complex and based on numerous interacting variables.

The differences between the mosquito species are particularly notable. While we were unable to do so due to resource and time limitations, the difference between insecticide susceptibility between the two species may be explained by differences in the prevalence of resistance mutations. In addition, both mosquito species have differential optimal temperatures for survival [8,9,10,11,16,17,29]. It seems likely that these responses may also extend to susceptibility to insecticides. *Aedes aegypti* is considered more adapted to warmer, dryer climates than *Aedes albopictus*. While the data is not perfect, it appears that *Aedes albopictus* is more susceptible to deltamethrin at mid and high temperatures and may be to a lesser degree susceptible to permethrin. These results suggest that any temperature acclimation or adaptations may also play a role in susceptibility to insecticides at different temperatures.

While our mosquitoes were from a field derived population, in some cases we observed decreased mortality to deltamethrin relative to permethrin for both species (Figure 1 and Figure 2). The reason for this is unknown but suggests that there may be cross-resistance to similar classes of insecticides, even without exposure to them. However, all of the populations would be classified as resistant to both permethrin and deltamethrin, using the CDC guidelines [30]. This preliminary study suggests the need to test multiple mechanisms of potential resistance, as well as environmental parameters, to obtain a greater understanding of the ability to control mosquitoes in different situations and environments.

The main method used to control the spread of vector-borne diseases is with the use of insecticides [22]. Efficacy of insecticides has historically been focused on evolution of resistance through the increased prevalence of resistance genes [3,4,6,7,9,18]. However, our data suggest that environmental factors may play a significant role in efficacy of insecticide control. Studies that examine efficacy of insecticides must consider these environmental influences, such as temperature, in order to reliably predict the efficacy of control efforts. In addition, our results highlight that public health officials must consider other environmental factors, such as temperature, when identifying the appropriate insecticide to use [31]. Our data suggest that during the hottest months, permethrin may be more effective than deltamethrin, but during cooler months there may not be any difference. Additional environmental variables and insecticides should be tested to produce a more robust recommendation. Additionally, alternative control measures should be considered and incorporated in control efforts to avoid time periods, such as cooler periods of the year, when insecticides may not be as effective.

## 4. Materials and Methods

### 4.1. Mosquito Population

F3 generation laboratory colonies of *A. aegypti* and *A*. *albopictus* eggs originating from McAllen, TX, were used to conduct the insecticide susceptibility experiments. Due to a lack of mosquitoes, F4 generation mosquitoes of *A. aegypti* were used to conduct low temperature trials. Eggs were hatched simultaneously in 1 g of nutrient broth and 1 L of deionized water. The solution was aerated for ½ hour prior to egg submersion, and eggs were then left submerged for 24 h. Larvae were reared at of 23 °C ± 2 °C; 75% RH, with natural light in trays with 1 L of deionized water. Larval densities were limited to 200 larvae to prevent overcrowding. Larvae were fed once every two days with 0.20 g of liver powder or 0.20 g ground fish flakes Tetra Color Tropical flakes© (altered each feeding period) until pupation.

Pupae were collected and placed in environmental chambers in cages measuring 31 cm × 31 cm × 31 cm [32] at three temperature settings to replicate conditions in the Rio Grande Valley in the summer (high), spring/fall (mid), or winter (low). Adult mosquitoes were kept in chambers with 75% RH and a 16 h:8 h light:dark cycle. Temperatures started to increase at 6 am and started to decline at 6 pm. These temperatures consisted of settings of 36 °C ± 2 °C (day) and 24.6 °C ± 2 °C (night) (high), 30.55 °C ± 2 °C (day) and 19 °C ± 2 °C (night) (mid), and 22.58 °C ± 2 °C (day) and 11 °C ± 2 °C (night) (low). Temperatures were set according to the City of McAllen TX U.S. climate data, reflecting the average temperatures for the years of 2007–2019 [33]. Inside the environmental chambers, adult mosquitoes were provided with sugar water and distilled water *ad libitum* prior to insecticide trials.

### 4.2. Bottle Bioassay Preparation

The CDC bottle bioassay [6,30] guidelines were used to prepare 250 mL Wheaton bottles for insecticide trials, with acetone used as a control. In brief, active ingredient insecticides were provided by the CDC to create stock solutions. These ingredients consisted of 2.15 mg permethrin or 0.0375 mg deltamethrin. These were combined with 50 mL of acetone to create stock solutions, which were refrigerated until use. Preparation of insecticide treated bottles consisted of taking 1 mL from the stock solution or control (pure acetone) and adding it to the interior of the bottles. Bottles were then rolled back and forth to ensure even coating along the entire interior of the bottle. Excess acetone was allowed to evaporate, and all bottles were capped then stored for 24 h prior to testing. Trials were conducted for three insecticides (including acetone control), three temperature regimes, and two species. Three replicates of each treatment and control were tested. Wheaton 250 mL bottles were coated with the insecticide (or control) for each species and temperature combination.

### 4.3. Insecticide Susceptibility Test

A total of 25 five- to ten-day old female mosquitoes from the environmental chambers were placed in each bottle bioassay for testing. Females were removed from the cage with an aspirator, briefly knocked down in a freezer for sorting, and then allowed to recuperate in a plastic vial for 10 min prior to being tested. Females were placed inside each bottle to monitor the mortality. Mortality was monitored at 15 m intervals at eight different time points for a total of 2 h. Center for Disease Control and Prevention guidelines were used to count “dead” mosquitoes [30]. If mosquitoes were knocked down, did not display the ability to mobilize, or were unable to fly, they were considered as deceased. Mosquitoes that retained the ability to move or fly reliably were considered alive. The diagnostic time (DT) of 30 m is used by the CDC to assess susceptibility in mosquito populations [30].

## 5. Data Analysis

Mortality rates were calculated at the DT and at the final time (120 m) by dividing the total number of dead mosquitoes by the starting number of mosquitoes. These rates were used as the response variable in all statistical analysis. Statistical analyses were conducted using JMP version 13 [34]. Multiple ANOVA analyses were conducted using each treatment option (insecticide, temperature, and mosquito species) as explanatory variables. One trial from *A. albopictus* at high temperatures and one trial of *A. albopictus* from mid temperatures were removed from the study due to incorrect preparation of the bottle assays. Insignificant interactions were removed from statistical analysis to avoid conflating the degrees of freedom. A post hoc Tukey–Kramer HSD test was conducted for pairwise comparisons between the treatments.

## Figures and Tables

**Table 1:** Statistical results for both the DT analysis and 120 m analysis.

Treatment	df	Diagnostic Time (30 m)		Final Time (120 m)	
		F Ratio	*p* Value	F Ratio	*p* Value
Temperature	2	40.0163	<0.0001	104.0659	<0.0001
Species	1	232.6249	<0.0001	74.5696	<0.0001
Insecticide	2	253.2861	<0.0001	1738.461	<0.0001
Temperature*Species	2	9.6182	0.0005	2.3748	0.1083
Temperature*Insecticide	4	26.1255	<0.0001	57.6673	<0.0001
Species*Insecticide	2	60.6902	<0.0001	41.6820	<0.0001
Species*Insecticide*Temperature	4	45.7056	<0.0001	10.8605	<0.0001

**Table 2:** Diagnostic and Final Time mean mortality rates.

Variables		Mortality at DT (120 m) ± Standard Error	Mortality at 120 m ± Standard Error
Temperature	Low	22.67% ± 8.18%	40.22% ± 9.00%
	Mid	28% ± 7.81%	56% ± 10.91%
	High	40.7% ± 9.20%	60% ± 11.37%
Species	*Ae. aegypti*	17.93% ± 4.68%	47.70% ± 7.83%
	*Ae. albopictus*	43.68% ± 8.07%	56.32% ± 9.32%
Insecticide	permethrin	57.5% ± 8.41%	93.75% ± 2.83%
	deltamethrin	36.44% ± 7.47%	66.44% ± 6.75%

**Table 3:** Treatment comparisons with significantly different treatment indicated (controls excluded).

Mosquito Species	Insecticide	Temperature	Diagnostic Time (30 m)		Final Time (120 m)	
			Avg. Mortality	Sig Different	Avg. Mortality	Sig Different
*Aedes albopictus*	Permethrin	Low	96.0% ± 0%	a	100%	a
		Mid	70.0% ± 18.0%	b	92.0% ± 8.0%	a
		High	70.0% ± 10.0%	b	100%	a
	Deltamethrin	Low	16.0% ± 2.3%	cd	44.0% ± 2.3%	c
		Mid	76.0% ± 2.3%	b	97.3% ± 2.7%	a
		High	82.7% ± 1.3%	ab	100%	a
*Aedes aegypti*	Permethrin	Low	16.0% ± 6.9%	cd	73.3% ± 4.8%	b
		Mid	21.3% ± 2.7%	c	98.7% ± 1.3%	a
		High	80.0% ± 4.0%	ab	100%	a
	Deltamethrin	Low	8.0% ± 2.3%	cd	24.0% ± 2.3%	d
		Mid	14.7% ± 2.7%	cd	60.0% ± 4.6%	bc
		High	21.3% ± 1.3%	c	73.3% ± 5.8%	b

## Data Availability

Data for this study is available through Christopher Vitek.
